# Medical Politics

**Published:** 1891-10-24

**Authors:** 


### The Hospital

A Weekly Journal of

Science, Medicine, Nursing, and Philanthropy.

*For Hospitals, Asylums, Infirmaries, Dispensaries, Medical Practitioners, Students, Nurses, and the Charitable Public.*

Vol. XI.—No. 265.] [Oct. 24, 1891.

### Medical Politics.

As a profession medicine is very much in the habit of consuming its own smoke. Doctors have a reputation, not altogether undeserved, for being somewhat quarrelsome among themselves. It matters little how many beams a particular medical man may have in his own eye, he is always most painfully solicitous about the motes which happen to be in the eye of his professional neighbour. This is only saying that there is a good deal of unregenerate human nature in the doctor's profession. But, at least, we do not habitually shriek aloud to the world at large.

There are two sorts of medical politicians in the little world of medicine; those who consider that that theirs is the best of all possible professions; and those who, on the other hand, regard it as one of the worst. The former class are usually prosperous and honoured; the latter may sometimes be honoured, though they are generally by no means prosperous. The rich and the famous among medical men are irritated by what they consider the undignified fractiousness of their humbler professional brethren; the less prosperous doctors, on their part, are exasperated by what they regard as the affected superiority and unsympathetic coldness with which they are treated by their more eminent confrères.

Whilst it need not be denied that there are in each case some grounds for these opposed opinions and feelings, it is pretty evident to the dispassionate observer that the faults on both sides are by no means preternaturally great. The struggling general practitioner may fret and fume a good deal at his hard lot, but his fretting and fuming ought not to be regarded as a disagreeable impairment of the dignity of his consultant brother. On the contrary, the consultant, whose lines have fallen to him in places so much more pleasant, ought to remember that it might have happened to him also to be a struggling general practitioner; and remembering this, his fellow-feeling should be wondrous kind. The corner house doctor on his part might profitably bear in mind that it is not given to every soldier in the army, but only to one here and there, to be a conspicuous leader; and seeing that this is the inevitable law of things he should clear his eye of jaundice and purge his soul of envy. Consultants there must be: let the hard-worked and ill-paid practitioner make the best use he can of their special skill. Above all let him be thankful that they are, as a class, capable in their work and honourable in their conduct. They present to the outside world a professional front which deserves and receives a very considerable measure of public confidence and admiration.

The profession is mildly agitated just now about a kind of medical general election. It threatens to be a very sober, possibly even a dull affair. Truth to say, there is little to excite enthusiasm on the one hand or opposition on the other. The election will have small practical result for the bulk of the profession. It will make nobody any richer and nobody any poorer; it will carry nobody up to a professional heaven and sink nobody down to an unprofessional hades. The candidates are eminently sober, respectable, and safe. There is not a fire-brand among them, not one who would willingly set the contents of even a six-ounce mixture on fire, much less the Thames. The medical profession, indeed, is not the profession for mèn of genius; doctors do not understand genius; they are afraid of it. There are two or three leading practitioners who might be named who would have been men of genius if they had gone into literature, or even to the Church or the Bar. But they have allowed themselves to be put into the hydraulic press of severe medical precision, with the result that each of them is now a square solid block of professional propriety of the most approved and strictly conventional type.

But the members of the Medical Council have their excellent qualities, and they care something for the profession, though that something be not very much. The general body of practitioners expect them to make two or three reforms; and it is as well that those members who are seeking re-election at the hands of the members of the profession should be asked to pledge themselves to the most urgent of those reforms. There are two facts connected with the entrance of young men to the profession which ought to be immediately looked to. The first of these is the initial examination for the status of a medical student, and the second is the date of the final examination for a State regulated licence to practise. At the present time a boy of any age may begin the study of medicine provided he can pass the examination; and the examination is so limited in extent and severity that any educated and intelligent boy of fourteen, who has been specially prepared for it, can pass it without difficulty.

Now, in our judgment, no boy who is intended for the practice of medicine should close the period of his general education before the age of eighteen; and no boy should begin the special studies proper to medical science who is not eighteen years old or more. The medical profession as a body exhibits many grave faults, which can be traced to the defective general education of the vast majority of its members. Self-respect, self-reliance, and broad intelligence are conspicuously deficient in large numbers of fairly competent medical men. They follow leaders, like sheep, in the most humiliating way. But they ought to be men of education and independent thought, not a mere illiterate multitude. The very spectacle we now see of an election of members to the Medical Council, carried on with a dulness which is absolutely deadly, shows how little independence of judgment and fresh originality of mind there is in the profession as a whole. Nothing would tend to drive away this deadly dulness so much as a broad and liberal general education. Youths designated for the medical profession should know something of literature, that life-enkindling spirit; something also of history, and not only of the stirring history of ancient Greece and Rome, but of the still more moving story of our own country; of the Danes and of the Saxons; of the Plantagenets, the Tudors, and the Stuarts; of Cromwell and of Milton; of Burke and of Wellington; and of the many other noble and striking figures whose deeds make an immortal epic of our island story. Philosophy, too, has its claims, and general culture.

The boy who has left school and been put to the minutiæ of medical study before the fires of his general intellectual life have been kindled, can hardly ever become what may be called a truly great man, and can only with infinite difficulty be developed into even a truly great doctor. As a man he is practically extinguished, and in many cases only survives as a dull and half dead piece of human mechanism. Poor fellow! He has never known what it is to live!

The other suggestion, that the elected members of the Medical Council should strive to raise the age of medical licensing to twenty-four instead of leaving it at twenty-one, where it now stands, is of decided importance. It has been frequently urged in these columns that to turn a young man loose at the age of twenty-one to practise medicine without control, on all and sundry, is ridiculous in itself and injurious to the public. We have before pointed out that no young man can be a fully-fledged clergyman until he is twenty-four years of age, and that very few clergymen indeed have the uncontrolled charge of parishes before they are thirty; and even that is quite early enough. Certainly the public interest demands that young medical men should not be licensed to practice at all before the age of twenty-four; and they should by no means be permitted to practise, except under the control of their seniors, until they are at least twenty-six or twenty-seven. It is very desirable that those gentlemen who seek election to the Medical Council at the hands of the great body of the profession should pledge themselves to push forward these urgent practical reforms with all speed.

There are three candidates for election to the Council who have thrown in their lot together; they are Sir Walter Foster, of Birmingham; Mr. Claudius Galen Wheelhouse, of Leeds; and Dr. James Grey Glover, of London. Those gentlemen have already represented the profession in the Council for five years. It has never been charged against them that they have been disloyal to their pledges, or to the interests of the great body of practitioners. On the contrary, each of them has done what was possible, with strictly limited opportunities, to push forward practical reforms and to conserve the rights of the electors. There is no reason whatever why those gentlemen should not be re-elected for a further period of five years; there are many reasons why they should. For our part we very cordially recommend them to the good will of our medical readers. The occasion, as we have said, is not one for any very perfervid enthusiasm; still it is eminently desirable that our elected representatives be men of weight and worth; and such, undoubtedly, are the three candidates whose names we have ventured to commend to the profession.

